# A novel founder variant in *BEST1* gene causing autosomal recessive bestrophinopathy

**DOI:** 10.1186/s13023-025-03813-1

**Published:** 2025-05-25

**Authors:** Nagham Maher Elbagoury, Caroline Atef Tawfik, Asmaa Fawzy Abdel-Aleem, Heba Mahmoud Fathy, Dina Nabil Baddar, Mona Lotfi Essawi

**Affiliations:** 1 Department of Medical Molecular Genetics, Human Genetics and Genome Research Institute, National Research Centre, Cairo, 12311 Egypt; 2 Center of Excellence for Human Genetics, National Research Centre, Cairo, Egypt; 3 Department of Ophthalmology, Ain Shams University, Cairo, Egypt; 4 Watany Eye Hospital, Cairo, Egypt; 5 Research Institute of Ophthalmology, Giza, Egypt

**Keywords:** Africa, Autosomal recessive bestrophinopathy, *BEST1* gene, Egypt, Founder effect, Missense variant, Novel variant

## Abstract

**Background:**

Autosomal recessive bestrophinopathy (ARB) is a rare retinal dystrophy caused by homozygous or compound heterozygous null variants in the *BEST1* gene. Clinically, ARB presents with variable features including central visual impairment, global photoreceptor dysfunction (as indicated by abnormal full-field ERG), and a significantly reduced electro-oculogram (EOG) light rise, a hallmark of bestrophinopathy. Fundus examination reveals widespread retinal pigment epithelial (RPE) disturbance, vitelliform deposits in the posterior pole (more clearly visualized with fundus autofluorescence), and macular fluid accumulation. Angle-closure glaucoma, secondary to anterior chamber dysgenesis, is a potential complication. This work aims at documenting the founder effect of a novel variant in the *BEST1* gene causing autosomal recessive bestrophinopathy and determining its variable clinical features.

**Methods:**

Twelve members of nine unrelated, consanguineous Egyptian families with a history of impaired central vision underwent comprehensive ophthalmological examination, fundus color photography, fundus autofluorescence (FAF), spectral-domain optical coherence tomography (SD-OCT) of the macula, and electrophysiological studies. Variant screening of coding exons of the *BEST1* gene and some flanking regions was performed using the Sanger sequencing technique. The pathogenicity of the variants was tested using different in silico functional analysis tools.

**Results:**

The clinical examination and investigations confirmed the ARB phenotype. All twelve patients exhibited (c.365 G > C, p. Arg122Pro) a novel *BEST1* gene variant in a homozygous form. On top of the classical retinal phenotype of ARB, some patients had other ocular associations: four patients were found to have angle-closure glaucoma, one patient had associated corneal dystrophy, one developed a macular hole, and one patient developed uveitis.

**Conclusion:**

The identification of the same, novel homozygous *BEST1* missense variant in twelve patients from nine unrelated, consanguineous families of Egyptian origin, suggests a founder effect. Angle-closure glaucoma was the most commonly associated ocular abnormality (30%). Our finding expands the molecular spectrum of ARB-associated variants, and identification of this founder variant can simplify genetic testing in the presence of limited resources and lead to better counseling.

## Background

The bestrophin-1 gene (*BEST1*, also known as *VMD2*) is a member of the bestrophin family of anion channels including *BEST2*, *BEST3*, and *BEST4* [1]. *BEST1* is located on the long arm of chromosome 11 (11q12.3) that encodes for bestrophin-1; a transmembrane protein consisting of 585 amino acids. The protein is situated on the basolateral membrane of retinal pigment epithelial (RPE) cells [2, 3]. The function of bestrophin-1 within the RPE is not fully understood yet, but it has been hypothesized to function as a Ca^2+^-activated Cl^−^ channel [4], a regulator of voltage-gated Ca^2+^ channels [5], and an HCO3^−^ channel [6]. The protein consists of six domains, four of which are transmembrane (first, second, fifth, and sixth domains) whereas the third and fourth domains are cytoplasmic ones [7]. Variants in *BEST1* thereby have an impact on RPE metabolism and subsequently outer retinal function.

*BEST1* gene variants have been associated with a variable spectrum of ocular presentations [8]. The first disease reported by *BEST1* sequence variants was Best vitelliform macular dystrophy (BVMD) [2], a retinal dystrophy characterized by bilateral involvement of the maculae with yellowish egg yolk-like lesions. *BEST1* variants are also associated with other disease entities that exhibit characteristic clinical heterogeneity. The diseases include adult-onset vitelliform macular dystrophy (AOVMD) [9], autosomal dominant vitreo-retinochoroidopathy (ADVIRC) [10], retinitis pigmentosa (RP) [11], the microcornea, retinal dystrophy, cataract, and posterior staphyloma (MRCS) syndrome [12], and autosomal recessive bestrophinopathy (ARB).

Schatz et al. described multifocal vitelliform deposits in two related patients who harbored compound heterozygous *BEST1* gene variants in 2006 [13]. It was not until 2008 that the term ‘ARB’ was coined by Burgess et al. [14]. Functionally, this disorder is characterized by a progressive, slow decline in central vision, absence of the electro-oculographic (EOG) light rise, and reduced full-field electroretinograms (ERGs). Phenotypically, fundi lack the typical egg yolk lesions distinctive of BVMD and instead demonstrate dispersed punctate flecks secondary to diffuse irregularity of RPE which appear as alternating areas of hypoautofluorescence and hyperautofluorescence within the posterior pole on FAF. Patients often present with fluid collection within and/or beneath the neurosensory retina involving the macular area with potential expansion beyond vascular arcades. Patients often exhibit hypermetropia and may have shallow anterior chambers which can predispose to angle-closure glaucoma [14]. ARB is hypothesized to be caused by the complete absence of bestrophin-1 protein within RPE cells in humans, thereby considered as the null phenotype of *BEST1* [15]. It has been reported either in homozygous or compound heterozygous variants in the *BEST1* gene [6, 14]. The onset can occur as early as the first two decades or remain undetected until the fifth decade, reflecting a wide range of variability. Overall, ARB represents approximately 4% of all inherited retinal diseases (IRDs) [16].

In this study, we aim to determine the founder effect of a novel variant in the *BEST1* gene present in twelve members from nine unrelated Egyptian families from Upper Egypt and document its variable clinical features.

## Methods

Twelve patients from nine unrelated, consanguineous, indigenous Egyptian families in Upper Egypt, all with a history of impaired vision, were included.

### Clinical examinations

All enrolled patients were diagnosed with ARB after a comprehensive ophthalmological examination encompassing recording of medical, ocular, and family histories, unaided distance visual acuity (UDVA), and corrected distance visual acuity (CDVA) testing using the Snellen chart, subjective refraction, ocular motility examination, intraocular pressure measurements using Goldmann applanation tonometry, as well as slit-lamp biomicroscopy including a dilated fundus examination.

Color fundus photography and fundus autofluorescence (FAF) using Heidelberg Retinal Angiogram (HRA-2; Heidelberg Engineering GmbH, Dossenheim, Germany) or ultrawide field fundus imaging using Optos California (Optos, Marlborough, MA, USA), spectral domain optical coherence tomography (SD-OCT) using Heidelberg Spectralis OCT (Heidelberg Engineering GmbH; Dossenheim, Germany) were performed. In addition, full-field electroretinogram (ERG) and electro-oculograms (EOG) were recorded using the RETIscan (Version 6.14.1.4, Roland Consult; Stasche & Finger GmbH, Brandenburg an der Havel, Germany) or LKC UTAS SunBurst (LKC Technologies, Inc., Gaithersburg, MD) according to the International Society for Clinical Electrophysiology (ISCEV) standards.

### Molecular genetic analysis

Blood samples were collected after obtaining written informed consent from the patients or their guardians. Three milliliters of peripheral blood were withdrawn from each patient on EDTA tubes. DNA was extracted by modified salting-out technique by *PAX* gene Blood DNA extraction kit (Qiagen, Germany) then the concentration and purity of the DNA were measured by NanoDrop spectrophotometer.

All DNA samples went through polymerase chain reaction (PCR) for amplification of the coding exons of the *BEST1* gene. This was followed by the purification of successfully amplified PCR fragments which were finally Sanger sequenced. The samples were injected into an automatic sequencer and data was analyzed using the 3500 ABI Prism DNA sequencing analysis software. Finch TV version 1.4.0 was used to display the data files obtained. Parental segregation analysis was only available to patient 2. Allele frequency was checked on 1000 genome and gnomAD (v4.1.0) control databases. Bioinformatic analysis was conducted to determine the pathogenicity of the detected variant using various in silico tools.

## Results

### Patient history

**Fig. 1:**
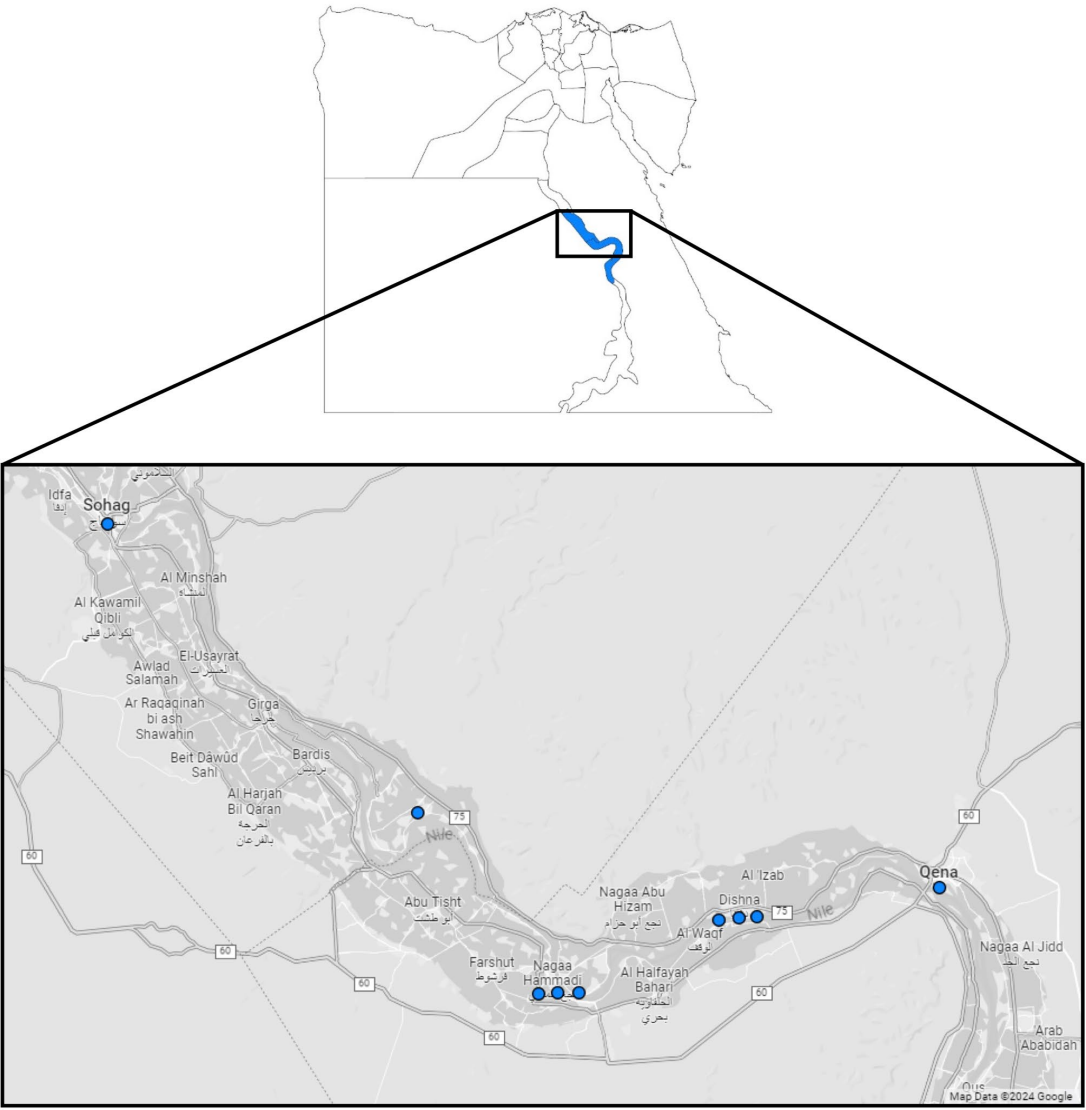
Geographical distribution of the 9 Upper Egypt families with ARB (blue circles)

Twelve patients (seven males, and five females) from nine unrelated families were examined in this study. Regarding age of onset, three patients were of childhood-onset (< 18 years) accounting for 25% of the cohort whereas the rest were of adult-onset. Age at first visit ranged from 10 years to 45 years with a mean of 27.8 years, and a median of 32 years. Nine patients had follow-up visits spanning over a few years ranging from 2 up to 9 years with a mean of 3.5 and a median of 2 follow-up years. The three remaining patients were followed up for less than a year, with each having one or two visits. Symptom presentation varied; some patients were asymptomatic and diagnosed incidentally, while others experienced stable or worsening central vision loss during the disease. All nine families were consanguineous and were geographically confined within two neighboring governorates in Upper Egypt, namely; Qena (one family originated from Qena municipal division, three families originated from Nagaa Hammadi division and three families originated from Dishna division) and Sohag (one family originated from El Kushh municipal division and another from Sohag division) (Fig. 1).

### Clinical findings

**Fig. 2:**
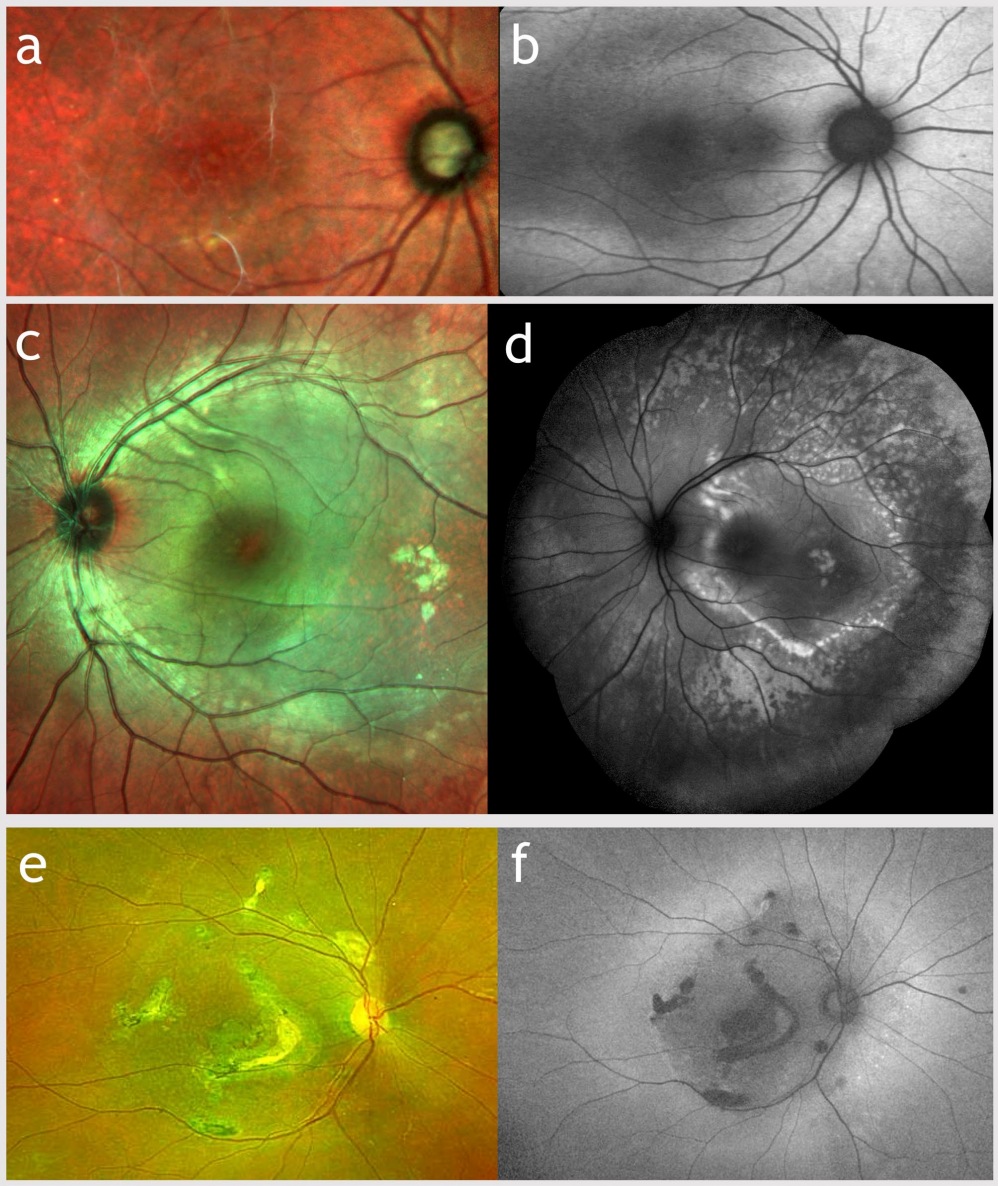
Different presentations and retinal findings in ARB as demonstrated on fundus photography and fundus autofluorescence (FAF). Patient 10 (**a**-**b**): subtle, continuous hyperautofluorescence area extending to the midperiphery without flecks; Patient 1 (**c**-**d**): multiple yellow confluent and discrete deposits in posterior pole, midperiphery and nasal to the disc; Patient 6 (**e**-**f**): multifocal fine vitelliform deposits associated with RPE atrophy, migration and subretinal gliosis involving the macula

Table 1 shows the clinical results of our data. Phenotypically, fundus changes had a wide range of heterogeneity (Fig. 2). Retinal findings included multiple, yellowish confluent, fleck-like lesions affecting the posterior pole and mid periphery (*n* = 6); multiple, small, fleck-like, non-confluent extending to the periphery (*n* = 2); subretinal fibrosis with midperipheral flecks (*n* = 2); continuous yellowish lesion without flecks extending to the periphery (*n* = 1); and macular RPE atrophic changes associated with multiple yellowish dots and patches of chorioretinal atrophy in midperiphery (*n* = 1).

**Table 1:** Detailed results of patients’ examination

Patient ID	Eye	Age (1st visit)/Sex	Family ID	Affected Family Members	Follow Up (y)	BCVA (1st visit)	BCVA (Follow up)	Refraction	Fundus findings	ERG	EOG Arden’s ratio	Association
1	OD OS	14/M	1	No	3	20/50 20/63	20/50 20/63	0.00/-0.75 × 170 +1.25/-0.25 × 175	Multiple fleck-like confluent yellow lesions in posterior pole & mid periphery	Rod & Cone dysfunction	1.3 1.2	N/A
2	OD OS	31/F	2	No	2	20/63 20/125	20/50 20/63	+ 1.00/-1.25 × 120 +4.50/-3.50 × 135	Multiple small, fleck-like, non-confluent dots extending to periphery	Isoelectric	1.1 1.1	Corneal dystrophy
3	OD OS	34/F	3	No	2	20/63 20/50	20/63 20/50	-0.75/-0.75 × 115 -2.50/-1.50 × 65	Two or more well-circumscribed lesions similar to multifocal BVMD	Rod & Cone dysfunction	1.1 1.1	Angle-closure glaucoma
4	OD OS	10/M	4	No	4	N/A	20/100 20/32	-0.75/-1.25 × 15 -0.25/-1.00 × 170	Multiple small & medium sized vitelliform deposits surrounding the posterior pole	N/A	N/A	Macular hole
5	OD OS	33/F	5	No	< 1	20/200 20/100	20/200 20/100	+ 1.00/-1.00 × 70 + 1.00/-0.75 × 130	Multiple yellow fleck-like confluent lesions in posterior pole & mid periphery	Rod & Cone dysfunction	1.0 1.2	N/A
6	OD OS	17/M	6	No	3	20/200 20/100	20/400 20/100	-4.00/-1.25 × 170 -5.00/-1.25 × 170	Parafoveal subretinal fibrosis alternating with RPE atrophy associated with midperipheral flecks	Rod & Cone dysfunction	1.5 1.3	Uveitis
7	OD OS	37/F	7	Yes (Brothers#8 & 9)	2	20/400 20/200	N/A	+0.75/-0.50 × 75 -1.00/-0.25 × 45	Macular RPE atrophic changes with multiple yellowish dots and patches of chorioretinal atrophy in midperiphery	Rod & Cone dysfunction	1.0 1.1	N/A
8	OD OS	36/M	7	Yes (Sister#7, brother#9)	9	20/100 20/125	20/200 20/160	+3.00/-0.50 × 110 +1.50/-3.00 × 90	N/A	N/A	N/A	Angle-closure glaucoma
9	OD OS	32/M	7	Yes (Sister#7, brother#8)	2	20/50 CF@50 cm	20/40 CF@50 cm	+ 1.00/-1.50 × 65 +1.00/-1.75 × 115	Multiple yellow fleck-like confluent lesions in posterior pole & mid periphery	N/A	N/A	Angle-closure glaucoma
10	OD OS	45/F	7	Yes (Sister#7, brothers#8&9)	5	20/40 20/40	20/63 20/50	+1.75/-1.50 × 85 +2.00/-0.50 × 105	Continuous yellowish lesion without flecks extending to periphery	N/A	N/A	Angle-closure glaucoma
11	OD OS	13/F	8	No	< 1	20/50 20/63	20/50 20/63	+ 3.75/-1.50 × 5 +3.50/-2.00 × 175	Multiple fleck-like confluent yellow lesions in posterior pole & mid periphery with areas of subretinal fibrosis	Rod & Cone dysfunction	N/A	N/A
12	OD OS	32/F	9	Yes	< 1	20/200 20/63	20/200 20/63	+ 2.50/-2.50 × 5 +1.50/-2.75 × 155	Two well-circumscribed lesions similar to multifocal BVMD	N/A	N/A	N/A

BCVA, best-corrected visual acuity; BVMD, Best vitelliform macular dystrophy; ERG, electroretinogram; F, female; M, Male; N/A, not available; OD, right eye; OS, left eye

SD-OCT revealed a wide range of signs in our studied eyes including subretinal fluid (17/24), vitelliform deposits (14/24), schitic spaces (14/24), elongated photoreceptors (14/24), RPE migration (3/24), and focal choroidal excavation (FCE) (5/24) (Table 2).

**Table 2:** Spectral-domain OCT parameters of 24 eyes with ARB

OCT Parameter	Frequency (*n*, %)
Vitelliform deposits Subretinal fluid Subfoveal Diffuse Intraretinal fluid Focal choroidal excavation (FCE) Outer retinal layer thickening RPE migration Subretinal gliosis	14, 58.3% 17, 70.8% 6, 25% 11, 45.8% 14, 58.3% 5, 20.83% 14, 58.3% 3, 12.5% 4, 16.67%

OCT, optical coherence tomography; FCE, focal choroidal excavation

ERG was performed in 7/12 patients revealing rod and cone dysfunction in 6/7 and was isoelectric in one patient 1/7. EOG was performed in 6/12 patients showing universally abnormal Arden’s ratio (range 1.0 to 1.5, mean 1.2).

**Fig. 3:**
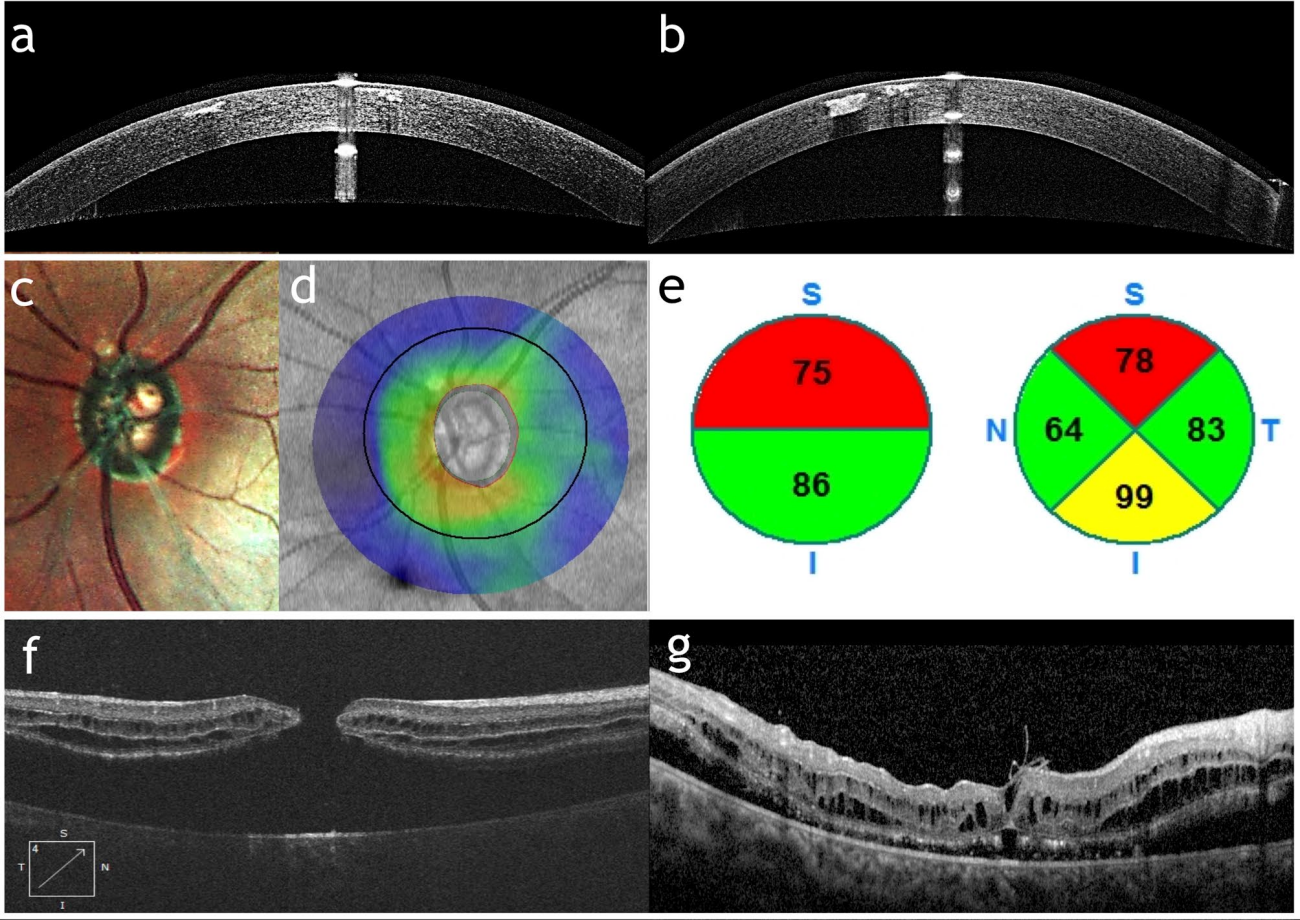
Associated ocular pathologies: Anterior Segment OCT of both eyes of patient 2 (**a**-**b**) demonstrating the superficial stromal hyperreflective deposits; Multicolor image (**c**), Retinal nerve fiber layer (RNFL) deviation Map (**d**) and pie charts of average RNFL thickness (**e**) of the left optic disc in patient 10 demonstrating glaucomatous thinning of neuroretinal rim; OCT B-scans of the right macula of patient 4 showing full-thickness macular hole before (**f**) and after (**g**) surgical repair

Additional ocular abnormalities were observed in our cohort. (Fig. 3) Angle closure glaucoma has been observed in 4/12 patients requiring topical anti-glaucoma medication in 4/8 eyes, surgical treatment in the form of phacoemulsification with gonioscopy-assisted transluminal trabeculotomy (GATT) in 2/8 eyes, filtering surgery in 2/8 eyes, and cycloablation in 1/8 eye.

Macular hole retinal detachment was observed in 1/12 patients with subsequent, successful surgical repair via an internal limiting membrane (ILM) flap.

Corneal dystrophy was observed in 1/12 patients. The dystrophic changes were recorded on anterior segment OCT (AS-OCT) as bilateral, dense, hyper reflective, organized material in the anterior corneal stroma sparing the epithelium and Bowman’s membrane. The patient was treated by phototherapeutic keratectomy (PTK).

Anterior uveitis was observed in 1/12 patients, with good response to topical corticosteroid treatment.

### Molecular results

**Fig. 4:**
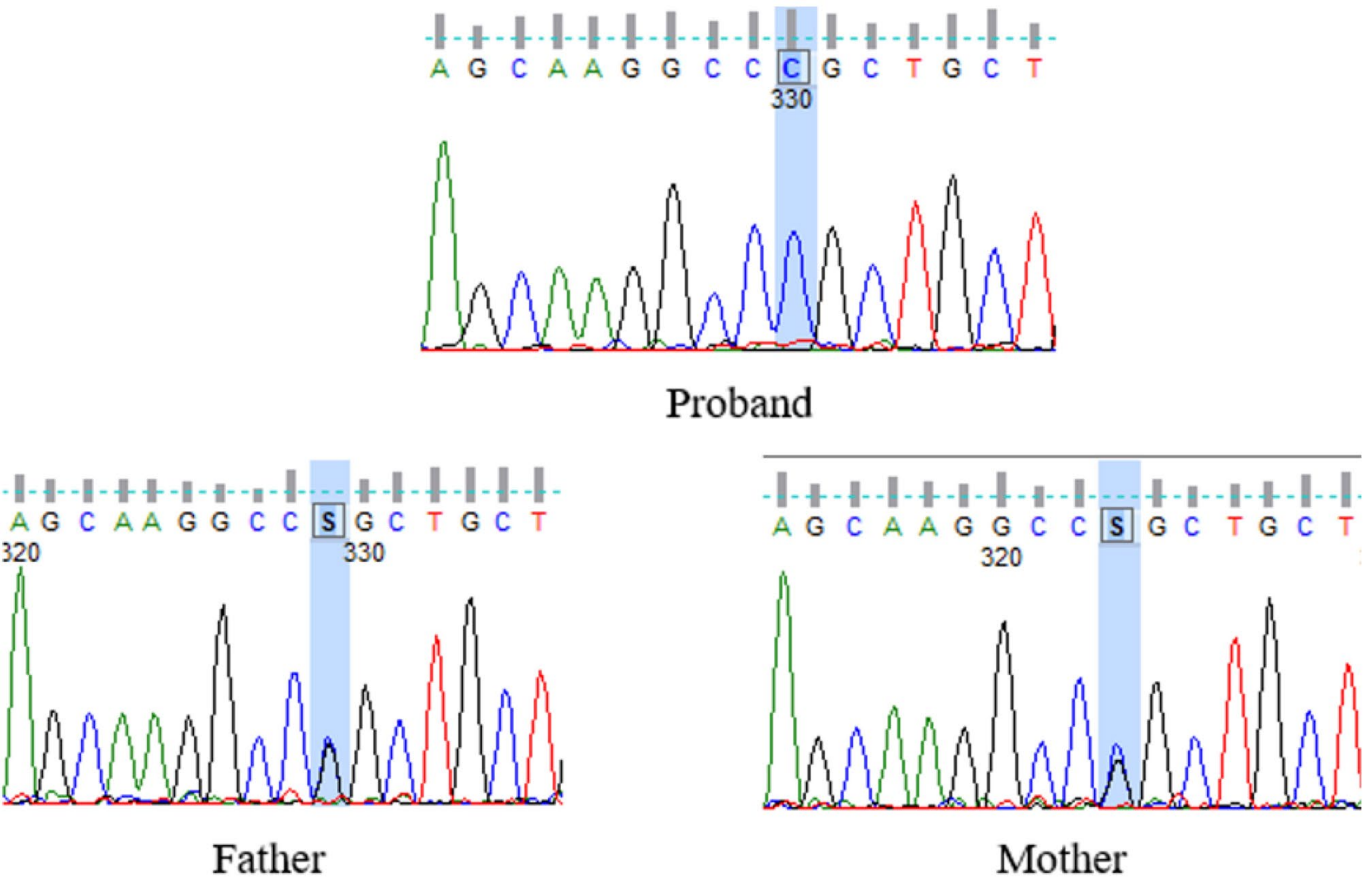
Chromatogram showing the detected variant (c.365G > C) in a homozygous form in the proband and in a heterozygous form in both parents of patient 2

Sanger sequencing of the coding exons of the *BEST1* gene (NM_004183.4) revealed the presence of a novel missense variant (c.365G > C, p.Arg122Pro) in all participating patients. The variant was submitted to ClinVar with allele ID SCV002499987. It was neither found on 1000 genome nor gnomAD (v4.1.0) control databases. Different in silico functional analysis tools were used to verify the pathogenicity of the variant. Parental segregation analysis was done in patient 2 where the parents were proved to be carriers of the variant (Fig. 4). Additionally, patient 6 underwent parallel genetic testing via a commercially available next-generation sequencing (NGS) gene panel covering 351 genes including an assessment of non-coding variants. The results showed no biallelic variants in any of the other genes tested.

The variant in question was a missense one where the amino acid arginine is replaced by proline at position 122 which is located 117 nucleotides upstream from the nearest splice site [17].

For pathogenicity assessment, Revel score was detected to be 0.97 which indicates a very high probability of the variant being pathogenic. This score is highly informative as it is calculated for missense variants based on the scoring of thirteen different tools namely: MutPred, FATHMM v2.3, VEST 3.0, PolyPhen-2, SIFT, PROVEAN, MutationAssessor, MutationTaster, LRT, GERP++, SiPhy, phyloPm and phasCons. CADD score, a tool for scoring the deleteriousness of single nucleotide variants, insertions, and deletions in the human genome, was calculated to be 31 indicating a deleterious effect caused by the variant.

**Fig. 5:**
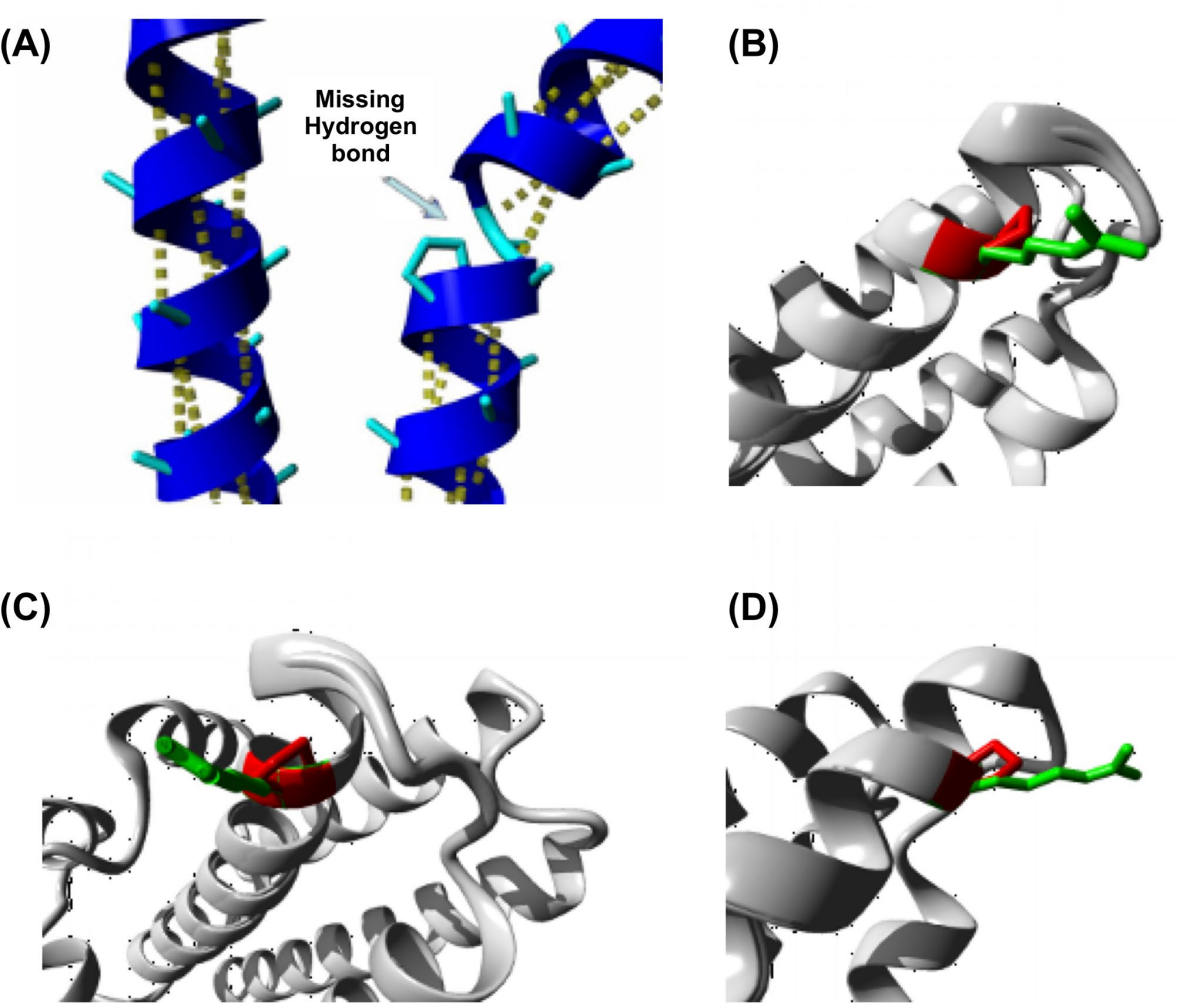
(**A**) Proline destabilize α-helix if not positioned at any of the first 3 positions of this helix. In this variant, disruption of the helix will take place leading to severe effects on the protein structure. (**B**) Closer view of the variant. The protein (grey color), the side chains of the wild-type (green color) and the mutant residue (red color) are shown. (**C**) Closer view of the variant from different angle. (**D**) Another view of the variant

Grantham scores are usually used to predict the difference between amino acids in their biochemical properties and hence the severity of a change. The Grantham score for the studied variant, where positively charged arginine was replaced by nonpolar proline, was 103. This moderately radical replacement likely alters protein function [18]. HOPE, another in silico functional analysis prediction tool, predicted the destabilization of α-helix [19] due to the replacement of arginine by proline as shown in Fig. 5. Additionally, this position is highly conserved among different species with a conservation score phyloP100: 6.501, by aligning the human genome to 99 vertebrate genome sequences.

## Discussion

ARB is a distinct type of retinal dystrophy caused by biallelic *BEST1* gene variants. The phenotype, however, is often characterized by a high degree of variability across patients [21]. Classically it is associated with scattered, subretinal, yellow deposits involving the posterior pole which may extend to the midperiphery. Marked FAF abnormalities are characteristic of these lesions, which are often accompanied by intra- or subretinal fluid. Hyperopia, short axial length, and angle closure glaucoma are additional common ocular findings. Marked reduction or absence of EOG light rise is a salient feature and is commonly associated with photoreceptor dysfunction on full-field ERG [14].

ARB is classified as a channelopathy, a group of diseases stemming from variations in cell membrane ion channels [20]. Bestrophin-1 protein comprises a transmembrane anion channel whose expression is mainly at the basolateral membrane of RPE [3]. To date, over 447 variants in the *BEST1* gene have been detected and 158 mutations have been correlated to bestrophinopathy cases. The vast majority of the reported variants are missense/nonsense followed by splice site variants, small deletions, small insertions, gross deletions, and only one mutation detected in the regulatory region as reported in the Human Gene Mutation Database (Professional 2024.1). Most of the described variants are aggregated near the transmembrane domains [7, 21]. Many of the disease-causing variants result in dysfunction of the chloride channels in RPE cells. The studied variant alters arginine at position 122, a highly conserved residue in the cytoplasmic region distant from the second transmembrane domain [22]. This is further verified by its Grantham Score of 103 pointing to a moderately radical replacement between amino acids. The change from arginine, which carries a positive charge, to proline, a nonpolar amino acid, leads to significant conformational changes and destabilizes the resulting helix as shown in Fig. 5.

In our study, we describe a cohort of twelve ARB patients of nine consanguineous, indigenous Egyptian families of Upper Egypt who all proved to share a novel missense variant in the *BEST1* gene. We postulated that this novel variant has a founder effect. Hence, we were faced with two challenges, firstly proving the pathogenicity of this variant, and secondly gathering evidence to support it being a founder variant.

In the first regard, we underwent segregation analysis in patient 2 whose parents were available, the variant did segregate with the disease where both parents proved to be heterozygous for the variant in question, and none of whom had any clinical manifestation of the disease. All in silico prediction tools we used supported its pathogenicity. Lastly, the NGS extensive retinal dystrophy panel done in patient 6 revealed no additional pathogenic variants, ruling out other genetic causes for the observed phenotype.

In the second regard, the concentration of all twelve patients within a 110-kilometer radius across the neighboring Upper Egyptian governorates of Sohag and Qena strengthens our hypothesis that the variant is a founder variant, stemming from a common ancestor. The potential for ancestral internal migration within the two governorates remains a valid consideration. Furthermore, the consanguineous nature of the nine families aligns with the documented high consanguinity levels, reaching 55.2% in Upper Egyptian rural regions, thus lending credence to our findings [23].

Throughout literature, proving a founder effect of a variant has taken place through haplotype analysis for patients by inspecting shared conserved adjacent polymorphic markers which was used in many studies related to various IRDs [24] including RP [25, 26], and ARB [27]. In the past few years, another approach was adopted by a nationwide genetic analysis conducted by Sharon et al. comprising 2420 families including 3413 patients with IRDs. The study came up with a conclusion that a variant had a founder effect if it was detected in at least three families of the same ethnicity [28]. In our study, nine families (threefold the presumed number of families required) from the same ethnicity and geographical region carry the same variant therefore a founder effect of this variant was proved. The observation of a novel variant exhibiting a founder effect in a relatively large cohort, juxtaposed with the disease’s infrequency, is noteworthy.

In our study, an attempt was made to establish a genotype-phenotype correlation through clinical documentation. However, significant inter- and intra-familial phenotypic variability, despite the shared variant, made this challenging. Specifically, both fundus and OCT findings demonstrated wide variability, a characteristic previously noted by Hufendiek et al. [29]. Furthermore, we observed several ocular associations, including angle closure glaucoma, macular hole, corneal dystrophy, and anterior uveitis, the relationship of which to the identified variant remains unclear. Notably, angle closure glaucoma is frequently associated with ARB due to anatomical factors such as narrow angles, reduced axial length, and hypermetropia [14, 30]. Furthermore, we observed several ocular associations, including angle closure glaucoma, macular hole, corneal dystrophy, and anterior uveitis, the relationship of which to the identified variant remains unclear. Corneal dystrophy has never been reported in association with ARB. Further, WES is needed to exclude variants in corneal dystrophy-related genes. Although anterior uveitis is a novel finding in ARB, the presence of aqueous flare and cells, indicative of retinal degeneration and photoreceptor death, is a recognized phenomenon in IRD patients [31].

The follow-up period ranged from 2 to 9 years. This longitudinal follow-up of patient 8 allowed us to delineate the disease progression attributable to this specific variant, yielding information useful for prognostic counseling of patients.

Identifying this founder variant offers a valuable opportunity to implement targeted diagnostic and screening strategies for ARB, which presents with significant phenotypic variability. Sequencing this recurrent variant is particularly advantageous and appealing socio-economically, especially in resource-limited countries and communities with limited access to genetic testing. Therefore, we recommend prioritizing this founder variant in ARB patients from the same geographical region to streamline genetic testing and enhance the speed and cost-effectiveness of diagnostic services.

To the best of our knowledge, this is the first clinical and molecular Egyptian study on a cohort of ARB patients. We characterized ARB in our cohort of patients and assessed the pathogenic and founder effect of c.365 G > C variant. This work may prove useful in testing patients manifesting ARB from Upper Egypt and specifically from Sohag and Qena governorates. Moreover, a large number of patients demonstrating a single variant can be ideal candidates for recruitment into clinical trials of any future treatment of ARB.

## Data Availability

The datasets used and analyzed during the current study are available from the corresponding author on reasonable request.

## Abbreviations

ADVIRC: Autosomal Dominant Vitreo-Retinochoroidopathy
AOVMD: Adult-Onset Vitelliform Macular Dystrophy
ARB: Autosomal Recessive Bestrophinopathy
AS-OCT: Anterior Segment Optical Coherence Tomography
BEST1: Bestrophin-1 gene
BVMD: Best Vitelliform Macular Dystrophy
CADD: Combined Annotation-Dependent Depletion
CDVA: Corrected Distance Visual Acuity
EOG: Electro-Oculogram
ERG: Electroretinogram
FAF: Fundus Autofluorescence
FCE: Focal Choroidal Excavation
GATT: Gonioscopy-Assisted Transluminal Trabeculotomy
HGMD: Human Gene Mutation Database
ILM: internal Limiting Membrane
IRD: Inherited Retinal diseases
MRCS: Microcornea, Retinal dystrophy, Cataract, and posterior staphyloma Syndrome
NGS: Next-Generation Sequencing
PCR: Polymerase Chain Reaction
PTK: Phototherapeutic Keratectomy
RNFL: Retinal Nerve Fiber Layer
RP: Retinitis Pigmentosa
RPE: Retinal Pigment Epithelium
SD-OCT: Spectral-Domain Optical Coherence Tomography
UDVA: Unaided Distance Visual Acuity
